# Comparison of Sample Preparation Methods for Multielements Analysis of Olive Oil by ICP-MS

**DOI:** 10.3390/mps2030072

**Published:** 2019-08-19

**Authors:** Fadwa Damak, Maki Asano, Koji Baba, Mohamed Ksibi, Kenji Tamura

**Affiliations:** 1 Tsukuba Life Science Innovation (T-LSI) Program, School of Integrative and Global Majors, University of Tsukuba, 1-1-1 Tennodai, Ibaraki 305-8577, Japan; 2 Institute of Agro-Environmental Sciences, NARO, 3-1-3 Kannondai Tsukuba, Ibaraki 305-0856, Japan; 3 Faculty of Life and Environmental Sciences, University of Tsukuba, Ibaraki 305-8572, Japan; 4 Environmental Engineering and Ecotechnology Laboratory (LGEET), National School of Engineers of Sfax (ENIS), University of Sfax, Route de Soukra Km 4 P.O. Box 1173, Sfax 3038, Tunisia

**Keywords:** multielements, olive oil, ICP-MS, microwave digestion, evaporation, ultrasound-assisted extraction

## Abstract

Elemental analysis of olive oils by Inductively Coupled Plasma Mass Spectrometry (ICP-MS) is challenging because of the high organic load in olive oil samples and the low analyte concentrations. However, conflicting operating procedures in the preparation of oils prior to analysis by ICP-MS have been reported to overcome these difficulties. This study compared three methods of inorganic elements’ extraction from olive oils: The two commonly used microwave-assisted, acid digestion, and liquid–liquid, ultrasound-assisted extraction methods; and an optimized method: The combined microwave digestion-evaporation. Overall, microwave digestion-based methods did not compare opportunely, and ultrasound-assisted extraction was found to provide the best accord between simplicity of use, detection limits and precision improvement. The detection limits were in the range of 0.3–160 µg·kg-^1^, 0.012–190 µg·kg^−1^ and 0.00061–1.5 µg·kg^−1^, while repeatabilities were in the range of 5–21%, 5.4–99% and 5.1–40% for the microwave digestion, the combined digestion-evaporation and the ultrasound assisted extraction, respectively. The ultrasound-assisted extraction is therefore recommended as a preparation method for olive oils prior to analysis by ICP-MS. The broader range of elements that can be accurately detected is expected to help increase the discriminatory power and performance of geographical traceability models.

## 1. Introduction

Determination of multielements in olive oils has gained an important place in the scientific community because of their increased application in geographical traceability studies. In fact, by detecting more elements, the probability of including elements with higher discriminatory power increases, improving the performance of the classification models [1]. But the extraction of metals from edible oils and elements’ determination by ICP-MS is difficult, and one of the most challenging analytical problems, due to the high viscosity of the matrix, leading to problems in leaching and dissolving [2]. Additionally, the edible oil matrix is characterized by: (i) A high organic load that increases the matrix effects and the possibility of polyatomic molecular interferences from elements like C, N and S. This high organic content can result in carbon deposition on the sampling cone and the loss of sensitivity, and (ii) an extremely low concentration of elements that makes it extremely prone to contamination during preparation [3]. To overcome these difficulties, different sample preparation procedures were proposed and applied and were thoroughly reviewed elsewhere [4]. A thorough compilation of the literature on multielement determinations in edible oils revealed that the microwave digestion with HNO_3_ and sometimes with addition of H_2_O_2_ is the most widely proposed, validated and used preparation method [5,6,7,8,9,10,11].

However, after reviewing in our last study, the results of metals concentrations in olive oils from studies published in the last decade, we reported for the first time in the literature a wide variability in these concentrations, even within the same country. We presumed that such variability could have been at least partially affected by the use of different and conflicting preparation methods [12]. This same observation was confirmed in another recently published study that also pointed out the limitations of the microwave digestion method. In fact, out of the initially analyzed twenty-nine elements, the authors could only detect seven elements. A high detection limit was amongst the most problematic factors leading to the elimination of almost three quarters of the elements of interest [13]. In fact, in the microwave-assisted digestion, up to 9 mL and 2 mL of concentrated HNO_3_ and H_2_O_2,_ respectively, are used to ensure total decomposition of the sample. The direct implications of the use of this considerable amount of concentrated acid are: (i) The corrosive nature of the digests to the ICP-MS components [14], and (ii) the high viscosity of the analytical solutions, which may result in matrix effects. The approach usually used to counter these effects is to make further dilutions (up to 250 folds dilution) to ensure a maximal residual acidity of 1% to 5% which is the usual acid medium of choice for most ICP-MS analyses [15]. While the actual concentration of some elements (e.g., Na, K, Mg, Ca, Fe and Zn) can reach the ppm level, that of other trace elements can be as low as the ppt level [16]. The further dilution of these extremely low concentrations would result in going below ICP-MS detection capabilities.

This situation highlights the need for a unique standardized preparation method in order to ensure consistency and allow rigorous comparisons between oils from different origins. This specific procedure also needs to improve the method’s detection limits, so more elements can be detected and then used as independent variables in the traceability model.

The first solution one can think about is to make sure to obtain lower residual acidity so that we reduce the volume of ultrapure water required to dilute the samples. This is possible by: (i) Dispelling the excess of the residual acid in the digest by evaporation to near dryness or (ii) decreasing the initially used amount of concentrated nitric acid. The latter possibility implies that the complete decomposition of the organic matrix may not be possible, so another type of method instead of the microwave-assisted digestion should be used: It is the liquid–liquid ultrasound-assisted extraction. This strategy has the benefit of speed and simplicity of application. It can also reduce the amount of reagents through the use of dilute acid solutions. Amelioration of the analytes’ recoveries in liquid–liquid extraction can be obtained through the use of ultrasonic energy [17].

The main objective of this study is to compare three methods of olive oil samples preparation prior to ICP-MS analysis: The two current microwave-assisted digestion, and ultrasound-assisted extraction of multielements methods and an optimized combined microwave digestion-evaporation method. These three methods are described in detail and their results compared to aid choosing the method that reaches lower detection limits and higher precision. The ultimate goal is to recognize the most robust preparation method which allows the reliable quantification by ICP-MS of multielements in olive oil samples.

The International Olive Council, whose mission is to develop and standardize chemical olive oil testing methods, has not yet specified a standard multielement determination method, contrary to other compounds (fatty acids, triacylglycerols, aliphatic alcohols, etc.), despite the proven importance of multielements in traceability issues. This has lead to the conflicting and wide variability of the reported results of multielements concentrations in olive oils presumably caused by using different preparation methods. The outcome of this study can then help researchers and analysts choose the best method, which is the ultrasound-assisted extraction for olive oil preparation for traceability purpose, to ensure consistency.

## 2. Materials and Methods

### 2.1. Equipment

A microwave oven equipped with a 10 position rotor and capable of delivering 1600 W of power (ETHOS 1600, Advanced Microwave Labstation, Milestone Inc., Sorisole, Italy) was used for closed-vessel digestion of samples and method blanks. Capped Teflon tubes were used to decompose samples and also evaporate them when necessary.

An ultrasonic bath capable of delivering 300 W of power and 55 °C of maximal temperature was used to carry out the liquid–liquid extraction of elements.

A commercial hotplate placed inside a fume hood was used to evaporate the residual acid to near dryness after digestion. The residual digests were kept in their initial recipients that were protected from the laboratory environment and potential air-borne contamination by a cone made by rolling a laboratory clean-tissue.

DigiTUBES that have an ultra-low leachable metal content, of class A tolerance at the 25 mL graduation (SCP Science, Montréal, QC, Canada), were used to collect samples after microwave digestion and dilute them to volume with ultrapure water.

A Milli-Q Integral 3 (Nihon Millipore, Tokyo, Japan) was used to prepare ultrapure water that was used to prepare all solutions, make dilutions and rinse material at all times during the experiment. 

The quantification of the elements was carried out with ICP-MS (Elan DRC-e (Perkin-Elmer SCIEX, Concord, ON, Canada) and NexION 300XX (Perkin-Elmer, Waltham, MA, USA)). Samples were introduced by means of a borosilicate glass nebulizer. ICP-MS is known to suffer from unwanted polyatomic isobaric interferences. Therefore, the elements were monitored in standard, kinetic energy discrimination (KED: He collision) and dynamic reaction cell (DRC: CH_4_ reaction) modes to check for and reduce polyatomic interferences, and the appropriate isotopes were used. Instrument performance was checked by a midrange continuing calibration verification (5 µg·L^−1^) every ten samples. Indium was used as an internal standard in all the three methods and added to all samples, calibration solutions, method blanks and solutions prepared for quality control to yield a concentration of 1 µg·L^−1^.

The operating conditions and parameters of ICP-MS are shown in Table 1 for each method.

### 2.2. Chemicals

61% electronic-grade (EL) nitric acid HNO_3_ (Kanto Chemicals, Tokyo, Japan), 30% atomic absorption spectrometry-grade hydrogen peroxide H_2_O_2_ (Wako Pure Chemical Industries, Osaka, Japan) for ultra-trace analysis, and 35% HCl (Wako Pure Chemical Industries, Osaka, Japan) were used to prepare samples. The influence of instrumental drift was corrected by using Indium (In) as internal standard prepared from 10 mg·L^−1^ CLISS-1 (SPEX CertiPrep, Metuchen, NJ, USA) to yield a concentration of 1 µg·L^−1^ in the samples, method blanks and calibration solutions.

### 2.3. Description of the Methods

#### 2.3.1. Microwave Digestion

Mineralization of olive oil samples was carried out according to the method described by Llorent-Martinez, Fernandez-de Cordova, Ortega-Barrales, and Ruiz-Medina with minor modifications [18]. The method consists of weighing 0.5 g of vigorously shaken sample and placing it directly into the digestion vessel, and adding 7 mL of HNO_3_ and 1 mL of H_2_O_2_. The vessels were placed in the microwave digestion system. The program of the microwave consisted of a ramp of 15 min to reach 200 °C and 1000 W, where the system was maintained for an additional 15 min. After being cooled to room temperature, samples were transferred into DigiTUBES and diluted to volume with ultrapure water. Samples were filtered using a 0.20 μm pore size syringe filter (Captiva econofilter, Agilent Technology, USA). Vessels were cleaned using the same microwave operating program after each digestion batch and successively rinsed with Milli-Q water.

#### 2.3.2. Optimization of the Combined Digestion-Evaporation 

##### Description of the Protocol

**Figure 1:**
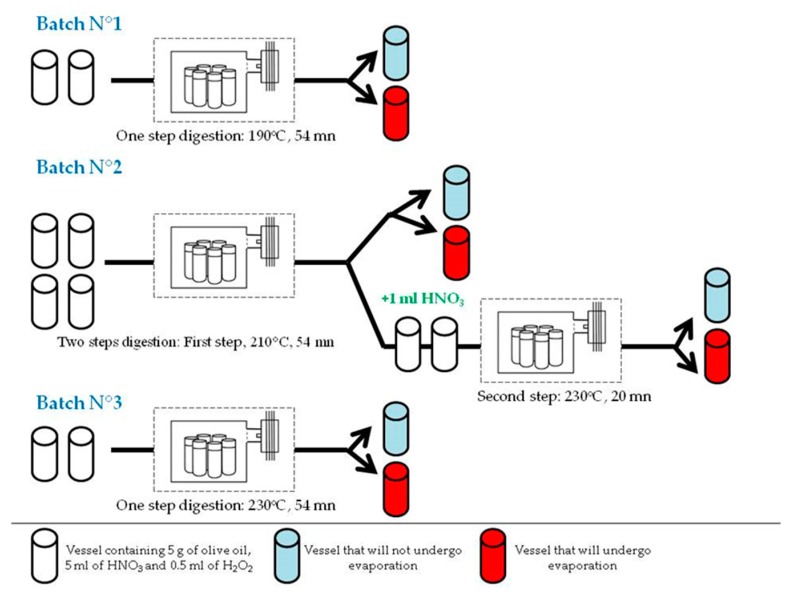
Scheme of optimization procedure for sample preparation prior to multielement determination by ICP-MS.

Three batches were designed with precise samples and reagents amounts specified and microwave digestion parameters defined according to the manufacturer’s recommendations (Figure 1). The first step of this method’s development was focused on optimizing the microwave working parameters (i.e., temperature (°C), time and number of digestion steps), and digestion vessels were prepared in duplicate in each batch so that one can be used to test the evaporation effect later on. 

The microwave program was chosen according to the manufacturer’s application note. The matrix that was the closest to that of olive oil was that of egg oil, so we have chosen the microwave program for egg oil digestion to digest samples of the first batch and then the temperature was increased by 20 °C at two consecutive times in each step of the programs of the following two batches. 

Following the digestion, the vessels that would not undergo evaporation were opened and their contents collected. The collected samples were then transferred to a DigiTUBE and their volume was adjusted to 25 mL. Samples were then centrifuged and diluted two times prior to ICP-MS analysis.

Table 2 shows the microwave program used for digesting the oil samples in each of the three batches. Each digestion cycle was followed by a 5 min ventilation cycle.

The other vessels, that were to undergo evaporation, were opened and uncapped. The bomb body was then placed on a hotplate until the residual digest volume became approximately equal to 1 mL (it takes around 30 min to reach that volume). After that, 20 mL of 1% HNO_3_ were gradually added to each vessel (10 mL at the first step that were left to dissolve the residual digest for about 5 min and then the total volume transferred to DigiTUBE and additional 10 mL added to wash the vessel). The volume was finally brought to 25 mL.

##### Selection Criteria

The quality of organic matrix decomposition was evaluated by determining the residual carbon content of the digested (RCCD) solutions. For the determination of RCCD, digested solutions were analyzed by ICP-MS using the semi-quantitative mode.

#### 2.3.3. Ultrasound-Assisted Extraction

The samples were prepared according to the method of Camin et al. with minor modifications [16]. Briefly, 10 g of olive oil sample was weighed into a 50 mL conical bottom polypropylene centrifuge tube and 10 mL of the extracting aqueous solution was added. The extracting water solution was prepared with: 1% HNO_3_, 0.2% HCl and 6.7% H_2_O_2_. The mixture was then vortex-shaken for 30 s and placed in a an ultrasonic cleaning bath (300 W, 26 °C) with a capacity of 30 L for 15 min to extract the inorganic elements from the oil to the aqueous solution. The mixture was then centrifuged (3500 *g* × 5 min) into separate the two phases. The upper oil layer was carefully aspired and discarded, the lower aqueous phase collected, and 5 mL of it were poured into a 15 mL conical bottom polypropylene centrifuge tube and subjected to ICP-MS analysis.

### 2.4. Quality Control for Performance Comparison

Validation of an analytical method refers to the precise characterization of the procedure so the most valid, well founded, reliable and precise measurement results can be acquired with it. It is one of the most critical steps in the process of introducing a new method into practice. The method validation must be conducted using the evaluation of at least the basic performance criteria which include: The estimation of the accuracy, the limits of detection and quantification, and the precision of the method under repeatable conditions [19]. In this work, the microwave digestion and the ultrasound-assisted extraction methods are already well established and validated methods. Only the combined digestion-evaporation method needed to be validated in case it showed better performance than the other two methods. Therefore, the parameters presented below will serve to compare the performance of the three methods suggested in this work.

#### 2.4.1. Limit of Detection (LOD) and Limit of Quantification (LOQ)

The values of LOD and LOQ are strongly related to measurement noise. LOD is the lowest concentration that can be measured (detected) by using a specified analytical procedure with statistical significance. LOQ is the lowest concentration that can be determined or quantified by using a specified analytical procedure with the established accuracy, precision and uncertainty.

LOD and LOQ of each element are calculated as three and ten times, respectively, for the standard deviation of the measurement of the specific element in ten independent method blank samples. Each method blank solution was prepared with the reagents used to prepare the samples and underwent the same analytical conditions as the samples [19].

#### 2.4.2. Precision (Relative Standard Deviation, RSD) 

It refers to the degree of variability among the independent measurements obtained by analysis of a specific sample by using a specific analytical method. Precision is usually expressed as a repeatability measurement of ten repeated determinations under the same conditions of a given sample on the same day, or it can be expressed as reproducibility measurement of ten different sample preparations on different days. In that case, it is expressed as a relative standard deviation [20]. For the microwave digestion method, precision assessment based on 10 independent samples was assessed herein by the mean RSDs of concentrations of the three replicates analyzed for each sample. The repeatability of the combined digestion-evaporation method was estimated based on seven independent replicates of a commercially available olive oil sample prepared and analyzed as described above. The repeatability of the ultrasound-assisted extraction was also calculated as a relative standard deviation of the concentrations of seven replicates of a commercial olive oil sample extracted and analyzed as explained above.

#### 2.4.3. Accuracy

The accuracy means the nearness of test results to the true value. The most used and common procedure of accuracy determination is based on the independent measurements of ten replicates of a certified reference material (CRM) and it is reported as the percent recovery of the known certified value. Sometimes, CRMs are not easily available and this may complicate the validation of analytical methods. In this study, the determination of the accuracy was hindered by the absence in the market of certified reference vegetable or edible oil that can matrix match that of olive oil. Other authors have previously tried to measure the accuracy by spike and recovery, using aqueous standard solutions, peanut butter CRM, or synthetic standard oil [6,16]. In another study, the authors validated a method of ultrasound-assisted extraction of multielements from olive oils by using a pomace sample and calculating the relative recovery of the elements from the same pomace sample prepared by a microwave digestion [13]. But we believe that these methods are not the best because they do not represent the real matrix of the edible oil and are likely to show low recovery mainly due to the high viscosity of the certified reference or standard materials and difficulties to obtain homogeneous mixtures. It is therefore of primordial importance to push certifying bodies to develop edible and specifically vegetable oils CRMs to facilitate the complete and effective validation of the proposed analytical methods of inorganic elements’ determination.

The accuracy of the microwave digestion method was checked in our previous study [12]. It was calculated by analyzing the multielement oil standard S23-100Y of 100 mg·kg^−1^ concentration (SPEXCertiPrep, Metuchen, NJ, USA) three times after appropriate dilutions, and calculating the recovery. The accuracy of the ultrasound-assisted extraction was evaluated by spiking three replicates of a commercial olive oil sample with a multielement standard solution at the level of 100 µg·L^−1^. The accuracy of the combined digestion-evaporation will not be determined, as this proposed method did not satisfy the primordial criteria of acceptable repeatability.

### 2.5. ICP-MS Calibration

Table 3 shows the calibration range of elements in olive oil samples in the three methods. External calibrations curves were built using a range of different mass concentrations prepared by a mixture of the following single-element and multi-element standard stock solutions: 10,000 mg·L^−1^ Ca (SPEXCertiPrep, Metuchen, NJ, USA), 1000 mg·L^−1^ Na (SPEXCertiPrep, Metuchen, NJ, USA), 1000 mg·L^−1^ Fe (SPEXCertiPrep, Metuchen, NJ, USA), 1000 mg·L^−1^ Mg (SPEXCertiPrep, Metuchen, NJ, USA) and 10 mg·L^−1^ XSTC-622B (SPEXCertiPrep, Metuchen, NJ, USA) (all in 5% HNO_3_).

Remark:

(i) XSTC-622B contains the following elements: Li, B, Na, Mg, Al, Si, K, Ca, Ti, V, Cr, Fe, Mn, Co, Ni, Cu, Zn, Ga, Ge, As, Se, Rb, Sr, Zr, Mo, Ag, Cd, Sn, Sb, Cs, Ba, W and Pb.

## 3. Results and Discussion

### 3.1. Microwave-Assisted Digestion

The Table 4 shows the results of performance criteria determinations for microwave-assisted digestion. The results shown are the ones collected during our previous study [12]. The linearity was very satisfactory with a linear regression coefficient greater than 0.999 for most of the elements. As for the LOD and LOQ, our results were significantly better than those reported in the original research [18] for the elements Fe, V and As, and relatively similar for the element Pb (Table 5). As previously stated by Konieczka, the values of LOD and LOQ are strongly related to measurement noise [19]. Therefore, the improvement of these values for Fe, V and As could be due to a lower background levels of these elements achieved by using the reaction mode of the Elan DRC-e ICP-MS, which decreases the measurement noise. Indeed, an Agilent 7500a that is not equipped with a collision/reaction cell was used in the study conducted by Llorent-Martinez et al. [18]. The accuracy was in the range 84–102% for almost all elements measured in the oil standard, except for Mg for which the accuracy was 66%.

### 3.2. Combined Digestion-Evaporation

#### 3.2.1. Carbon Content in Digest

Sample digestion or decomposition is an important step in analytical methods for the routine determination of chemical elements in foodstuffs. The particular degree to which decomposition is complete was assessed by measuring the RCCD. The study with a one-step digestion at 190 °C followed by evaporation on a hotplate showed the lowest RCCD values compared to those obtained by higher temperatures or a two-step digestion process (Table 6). Overall, all the vessels that underwent evaporation after the microwave-digestion step showed lower residual carbon content. This result was expected as the prolonged exposure of the sample to heat over time in its acidic environment increases its decomposition rate and the release of the volatile carbon to the atmosphere. As for vessels that did not undergo evaporation, we noticed that the residual carbon content increased as the temperature increased, and it exceeded the range when a two-step digestion process was employed. This result can be attributed to one of these causes: We presume that the high temperatures and pressures inside the closed vessels would have caused recalcitrant organic compounds’ formation to be difficult to decompose, leading to higher carbon content; or that the lower temperatures resulted in higher carbon content in reality but that caused lower sensitivities due to carbon deposition.

Efficient digestions should allow a complete decomposition of organic material, leading to low residual carbon contents [20]. The results presented in this study support the fact that digestion at 190 °C followed by evaporation confirms the observations made by Castro et al. [21]. Consequently, we will determine the performance criteria of this method; namely: LOD, LOQ and precision as a first step of the method’s development procedure.

#### 3.2.2. Performance Parameters of the Proposed Combined Digestion-Evaporation Method

The table below (Table 7) shows the results of the performance criteria determination for the proposed method.

##### Linearity

The ICP-MS analysis was calibrated using eleven external standards, including the blank. Although ICP-MS is well known for a wide linear dynamic range, we checked the linear regression coefficient of the calibration curves. Linearity is considered satisfactory if the coefficient exceeds 0.999 [15]. R^2^ was greater than 0.999 for Mg, Ca, V, Cr, Ni, Zn, As, Rb, Sr, Mo and Pb. The rest of the elements had lower coefficients, especially Fe which had the lowest one.

##### LOD and LOQ

When compared to the microwave digestion method, the combined digestion followed by evaporation method proposed here offers significantly better detection limits and consequently better quantification limits.

##### Precision (Repeatability)

The results of repeatability obtained were not satisfactory and showed high spread around the mean. Elements such as V, Fe, Rb and Mo had RSDs higher than 30%. V, Mn and As had RSDs higher than 50% (99% for As). Since RSD estimation was based on counts and not on concentrations, the RSD is sometimes not representative of the real spread; for example, in the case of As, counts varied between 0 and nine, which means the As was almost absent from the sample solutions, so an RSD of 99% is not representative of the real situation in the case of low-count elements. But Fe had high counts (*n* = 7: 4274 ± 1923) and still give an unsatisfactory RSD (45%).

### 3.3. Ultrasound-Assisted Extraction

Table 8 shows the results of the performance criteria determination for the ultrasound-assisted extraction method.

#### 3.3.1. Range of Linearity

The ICP-MS analysis was calibrated using twelve external standards, including the blank. Although ICP-MS is well known for a wide linear dynamic range, we checked the linear regression coefficient of the calibration curves. R^2^ obtained for most of the elements was satisfactory with some exceptions. Cu had the lowest coefficient (0.9931) and only the alkaline Rb, Sr and Ba had coefficients equal or higher than 0.999.

#### 3.3.2. LOD and LOQ

When compared to the microwave digestion method, the ultrasound-assisted extraction method offers significantly better detection limits and consequently better quantification limits. The sensitivity improvement varied from eight-fold for Rb to as high as 20,000 fold for Fe. The obtained LOD values were in the same range of those reported in previous studies on ultrasound-assisted extraction of elements from olive oils [13,16].

#### 3.3.3. Precision (Repeatability)

The results of repeatability obtained for the sonication method were very satisfactory compared to the two previous methods. Fourteen elements had RSDs lower than 13.4% whereas Ni, Cu and Zn had the least satisfactory RSDs (40%, 30% and 18% respectively) but that is still the best compared to the other methods.

#### 3.3.4. Accuracy

The accuracy was in the range 63%–136% for the determined elements measured in the spiked oil samples, which were more scattered than the accuracy results of the microwave digestion method. Overall, the ultrasound-assisted extraction sowed less recovery than the microwave digestion for most of the elements, as expected except for Na and Ba, but the results were generally deemed satisfying [17].

### 3.4. Comparison between the Three Methods

Based on the results presented above, the combined digestion-evaporation and the ultrasound-assisted extraction performed better than the microwave digestion as judged by the detection limits. So it is advantageous to replace the microwave digestion only by one of these two proposed methods to improve the detection limits and widen the range of the mass numbers that can be detected by the ICP-MS in the olive oil samples. Our results agree with those recently reported by Pošćić et al., stating that the microwave acid digestion does not allow measurement of elements present at very low concentrations even if the ICP-MS technique is used for detection [13]. This is because of the limited amount of sample that can be processed. The high pressure that develops during digestion owing to the high organic matter and high fatty acid content of the olive oil restrains the amount of sample allowed into the digestion vessel. Moreover, the residual acidity in the digests make it mandatory to dilute samples prior to ICP-MS analysis, which lowers the concentration of the elements, and may make them within the same range of the blank for some elements that are initially present at trace levels, and therefore make them undistinguishable from the uncertainty of the blank (i.e., very close or below the detection limit). Moreover, the same authors reported high detection limits obtained by the microwave digestion only, as they could detect only seven (Cu, K, Mg, Mn, P, Rb and S) out of the twenty-nine measured elements [13]. In another study comparing the performance of microwave digestion and simple dilution of wine samples prior to ICP-MS measurement, the authors reported that the detection limits for most elements in the digested samples were between two and 10 times higher than those for the diluted samples, and attributed these findings to the higher background in the method blanks of the digested samples. That higher background was due to the impurities in the reagents used to mineralize the samples and to possible contamination from the digestion vessels [22].

When compared together, the ultrasound-assisted extraction performed better than the combined microwave digestion-evaporation method, as judged by the detection limit and the precision expressed as repeatability. Those lower detection limits and the higher precision can be explained by the fact that the ultrasound-assisted extraction method uses fewer steps to extract the elements from the samples and to prepare the method blanks. On top of that, this preparation method exposes the samples to the laboratory environment for a shorter duration of time which means that it reduces the contamination risk. In fact, the dependable determination of elements at very low levels in organic materials is significantly determined by the ability to control and reduce contamination in the method blanks and sample solutions during sample preparation, because detection limits’ estimation is based on the variability of the blanks. This confirms the statement that an effective sample preparation method is the one that involves minimal preparation steps, since the risks of loss or contamination are then limited [20]. It remains important to say that to determine the accuracy of the preparation methods easily and efficiently, the use of vegetable oil CRM is recommended. This task is hindered at the moment by the absence of matrix matching CRMs. For this reason, it is extremely urgent to conduct future research to develop vegetable oil CRMs. This is crucial to help truly validate existent and future multielement determination methods, taking into account that, until now, most of the validations are based on spiking natural oils with natural or synthetic oils or butters, and recovery of the elements or calculations of the relative recovery, by comparison with another established method. It is worth noting that it is very challenging to obtain homogenous and representative samples with the currently available ultra-viscous oil or butter standards to reflect the reality of the vegetable oil matrices.

## 4. Conclusions

The accurate determination of multielements in olive oils is often limited by contamination encountered during sample preparation rather than sensitivity of the analytical technique. We believe that the ultrasound-assisted extraction of multielements from olive oil samples is preferred over the most commonly used microwave-assisted digestion procedure, because of the following advantages:  Lower detection limits, higher precision, less risk of contamination due to exposure to the laboratory surrounding environment, less time-consuming and simple sample preparation. Future research work should focus on developing edible oils’ CRMs, to aid in the validation of proposed analytical methods for high outcome-confidence, given the implication and importance of multielements in authentication, traceability and quality issues.

## Figures and Tables

**Table 1:** ICP-MS operating conditions.

Parameter	Method		
	Microwave Digestion	Digestion-Evaporation	Ultrasonic Extraction
Instrument	ELAN	NexION	NexION
ICP Rf power (W)	1100	1600	1600
Plasma Ar flow rate (L·min^–1^)	15	18	18
Auxiliary Ar flow rate (L·min^–1^)	1.30	1.20	1.20
Nebulizer (carrier gas) flow rate (L·min^–1^)	0.77	0.98	0.98
Sampler and skimmer cones	Nickel	Nickel	Nickel
lens voltage (Deflector voltage) (V)	7.5	−11.50	−11.50
Analog stage voltage (V)	−1700	−1800	−1800
Pulse stage voltage (V)	950	1100	1100
Discriminator threshold (V)	19	13	13
Quadrupole rod offset (V)	−1.5(STD), –7.5 (DRC)	0 or 0.50 (STD), –12 (KED), –7.5 (DRC)	0 or 0.50 (STD), –12 (KED), –7.5 (DRC)
Detector	Pulse	Pulse	Pulse
Speed of peristaltic pump (rpm)	20	20	20
Sweeps/reading	20	20	20
Replicate/reading	1	1	1
Replicates	3	3	3
Dwell time (ms)	50	50	50
Scan mode	Peak hopping	Peak hopping	Peak hopping
STD and KED: rejection parameter a and rejection parameter q	0, 0.25	0, 0.25	0, 0.25
DRC mode: CH_4_ reaction gas flow (L·min^–^1)	0.60	0.60	0.60
DRC mode: rejection parameter a and Rejection parameter q	0, 0.65	0, 0.65	0, 0.65
KED mode He reaction gas flow (L·min^–^1)	-	3.5	3.5

STD, standard mode; DRC, dynamic reaction cell mode; KED, kinetic energy discrimination mode.

**Table 2:** Operating program for the microwave system used for the three batches.

Batch N°	Step	Time (mn)	Power (W)	Temp 1 (°C)
1	1	2	1000	50
	2	1	0	30
	3	31	1000	190
	4	1	0	160
	5	6	1000	190
	6	13	1000	190
2	1	2	1000	70
	2	1	0	50
	3	31	1000	210
	4	1	0	180
	5	6	1000	210
	6	13	1000	210
3	1	2	1000	90
	2	1	0	70
	3	31	1000	230
	4	1	0	200
	5	6	1000	230
	6	13	1000	230

**Table 3:** Calibration range of elements in the three analytical methods’ analysis by ICP-MS.

	Solution Name (Concentrations in µg·L^−1^)					
Method	Na	Ca	Mg	Fe	XSTC-622B	N *
Digestion	-	-	-	-	0.01–50	8
Combined digestion-evaporation	0.05–100	0.025–50	0.01–20	0.01–20	0.005–10	11
Ultrasound-assisted extraction	0.05–50	0.025–25	0.01–10	0.01–10	0.005–5	12

* number of calibration points evenly distributed across the corresponding range.

**Table 4:** Results of determination of analytical procedure parameters (Microwave digestion at 200 °C, according to Llorent-Martinez et al., 2014 with minor modifications).

Element	Isotope	Operation Mode	LOD (µg·kg^−1)^	LOQ (µg ·kg ^−1)^	Repeatability (RSD %) *	Linearity (R^2^)	Accuracy (%)
Na	23	Standard	120	350	9.9	0.9998	84
Mg	24	Standard	160	470	12	0.9991	66
Fe	56	DRC (CH_4_)	120	390	13	0.9999	88
Zn	66	DRC (CH_4_)	110	360	16	0.9997	97
V	51	Standard	1.7	5.6	14	1.0000	97
Mn	55	Standard	6.0	20	20	1.0000	103
As	75	Standard	0.73	2.4	20	0.9990	
Rb	85	Standard	0.30	1.0	17	0.9996	
Sr	88	Standard	5.1	17	5	0.9999	
Ba	138	Standard	4.6	15	15	1.0000	102
Pb	208	Standard	6.9	23	21	1.0000	97

* based on the mean RSD of three replicates of 21 olive oil samples.

**Table 5:** Comparison of the limits of detection of the microwave digestion method between our results and those of the original research (for elements in common).

	LOD (µg·Kg^−1^)	
Element	Our Study	Original Study
Fe	120	600
V	1.7	15
As	0.73	15
Pb	6.9	4

**Table 6:** Counts at mass number 12 corresponding to the most abundant isotopes of carbon: A comparative method of residual carbon content estimation under different decomposition conditions.

	One Step						Two Steps	
	190 °C		210 °C		230 °C		210 °C then 230 °C	
Mass number	NE*	E**	NE	E	NE	E	NE	E
12	172,855,743	13,216,179	217,036,881	77,300,752	219,254,250	191,976,927	over range	76,931,314

NE, no evaporation; E, with evaporation.

**Table 7:** Results of determination of analytical procedure parameters (Microwave digestion at 190 °C followed by evaporation).

Element	Isotope	Mode	LOD (µg·kg^−1^)	LOQ (µg· kg^−1^)	Repeatability (RSD %)	Linearity (R^2^)
Na	23	He	13	45	5.4	0.9985
Mg	24	He	3.5	11	27	0.9991
Ca	44	CH_4_	190	640	6.6	0.9990
V	51	He	0.012	0.041	53	0.9996
Cr	52	CH_4_	0.47	1.6	25	0.9996
Mn	55	He	0.49	1.6	52	0.9989
Fe	56	He	5.8	19	45	0.9957
Ni	60	CH_4_	2.5	8.2	26	0.9998
Cu	63	He	2.3	7.7	16	0.9989
Zn	64	CH_4_	13	42	12	0.9997
As	75	He	0.093	0.31	99	0.9993
Rb	85	STD	0.036	0.12	38	1.000
Sr	88	STD	0.098	0.33	15	1.000
Mo	98	CH_4_	0.15	0.51	46	0.9999
Pb	208	STD	2.4	8.1	22	0.9999

He, Helium collision mode; CH_4_, CH_4_ dynamic reaction cell mode; STD, no gas standard mode; R^2^, linear regression coefficient of calibration for LOD and LOQ determinations.

**Table 8:** Results of determination of analytical procedure parameters for ultrasonic extraction of elements.

Element	Isotope	Mode	LOD (µg·kg^−1^)	LOQ (µg·kg^−1^)	Repeatability (RSD %)	Linearity (R^2^)	Accuracy (%)
Na	23	He	0.42	1.4	11	0.9985	136
Mg	24	He	0.11	0.35	11	0.9986	73
Ca	44	He	1.5	4.9	13	0.9811	
Ti	47	He	0.16	0.45	11	0.9977	
V	51	He	0.069	0.23	1.9	0.9975	
Cr	52	He	0.035	0.12	12	0.9987	64
Mn	55	He	0.0060	0.021	10	0.9979	67
Fe	56	He	0.14	0.47	5.1	0.9984	
Ni	60	CH_4_	0.18	0.60	40	0.9974	81
Cu	63	He	0.20	0.66	30	0.9931	80
Zn	66	He	0.077	0.26	18	0.9988	84
As	75	CH_4_	0.036	0.12	6.2	0.9987	
Rb	85	STD	0.00061	0.0021	8.9	0.9993	79
Sr	88	STD	0.0025	0.0085	9.1	0.9993	77
Mo	98	STD	0.0047	0.016	8.6	0.9970	78
Ba	138	STD	0.0014	0.0049	13	0.9990	133
Pb	208	STD	0.0035	0.012	13	0.9989	

He, Helium collision mode; CH_4_, CH_4_ dynamic reaction cell mode; STD, no gas standard mode; R^2^, linear regression coefficient of calibration for LOD and LOQ determinations.
